# Effect of Substrate Types on the Structure of Vertical Graphene Prepared by Plasma-Enhanced Chemical Vapor Deposition

**DOI:** 10.3390/nano11051268

**Published:** 2021-05-12

**Authors:** Siyi Xie, Junjie Huang, Yufeng Zhang, Weiwei Cai, Xueao Zhang

**Affiliations:** College of Physical Science and Technology, Xiamen University, Xiamen 361005, China; xiesiyi@stu.xmu.edu.cn (S.X.); huangjunjie@stu.xmu.edu.cn (J.H.); yufengzhang@xmu.edu.cn (Y.Z.)

**Keywords:** vertical graphene, electrical conductivity, morphology, atomic structure

## Abstract

Although the structure of vertical graphene (VG) is important for various applications, the growth mechanism of VG is not yet fully clear. Here, the impacts of electrical conductivity of substrate on the morphology and structure of VG prepared by plasma-enhanced chemical vapor deposition are studied by scanning electron microscopy and Raman spectroscopy. The results show that VG with greater thickness can be grown on substrate with better electrical conductivity in the same growth time. Even though longer deposition time leads to more VG, more defects might develop in VG, especially at the position furthest away from the substrates. The change of morphology and structure of VG is closely correlated with strength of electric field near the substrate surface, which offers a new approach for orderly growing of VG. The discoveries not only shed light on the growth mechanism of VG, but also are beneficial for promoting the applications of VG.

## 1. Introduction

Graphene is a two-dimensional carbon nanomaterial, in which carbon atoms form a hexagonal honeycomb lattice with sp² hybrid orbitals. The unique atomic structure of graphene leads to excellent electrical, optical, and mechanical properties, such as theoretically up to 2600 m^2^/g specific surface area [1], 97.7% light transmittance [2], 5300 W/mK thermal conductivity [3], 130 Gpa intrinsic strength [4], and carrier mobility at room temperature 15,000 cm^2^·V·s^−1^ [5], as well as observation of unusual physical phenomena, such as room temperature Hall effect [6], and room temperature ferromagnetism [7].

Recently, vertical graphene (VG) has attracted great interest from researchers due to its freestanding three-dimensional network structure and inheriting the excellent intrinsic properties of graphene [8,9,10,11]. Three-dimensional interconnected network structure and rich sharp edges endow VG with many excellent properties, such as high mechanical stability, large specific surface area, and sensitive sensing characteristics [12,13,14], which have great potential in various applications (e.g., energy storage, sensors, and thermal management) [15,16,17,18,19,20]. Plasma-enhanced chemical vapor deposition (PECVD) is the method most commonly used to synthesize VG [12,21,22,23]; PECVD can carry out chemical reactions at a relatively low temperature, which results in low thermal kinetic energy for all atoms/ions. The diffusion barrier may be reduced by the electric field-induced polarization effects that in turn will reduce the adhesion/bonding energy of carbon species to the substrate’s surface [24]. Meanwhile, the reactive gas molecules are decomposed into electrons and cations by the plasma, which will be influenced by the electric field in the deposition process. For example, a strong local electric field near the substrate will lead to a more orderly growth of graphene.

Compared to graphene films grown parallel to the substrate, the growth mechanism of VG is still unclear [23,24,25,26]. For example, the initial of nucleation is under debate: some reports claim that thermal stress causes the buffer layer to warp, resulting in nucleation sites [27], while other reports assert that the stress caused by lattice mismatch is the reason for the tear of the buffer layer and the formation of nucleation sites [24]. In addition, the different etching rates of amorphous carbon (a-C), sp^3^-C, and sp^2^-C by the etchant (mainly atomic hydrogen) are considered to play a key role in the structure of VG [25]. Moreover, the built-in electric field induced by the plasma also significantly affects the growth of VG [24,28]. In general, growing VG by PECVD is a complicated process, where the morphology, size of VG nanosheet, and crystallinity are strongly influenced by gas composition, pressure, growth time, substrate types, and substrate temperature [13]. Although these factors make it difficult to fully understand the exact growth mechanism of VG, researchers have proposed a possible three-step growth process including nucleation, vertical growth, and closure. First, nucleation sites are formed in the buffer layers, such as amorphous carbon or SiC, which usually is not correlated with the quality of VG. Then, active edges are generated due to lattice mismatch and deformation of the buffer layer induced by thermal energy. Due to temperature gradients, ion bombardment, lattice mismatch, and electric field near the substrate, the graphene grows upward along the active edges. In addition, carbon atoms can also be deposited on the surface of the graphene sheet and grow continuously along the sheet. When the material deposition and etching induced by plasma is balanced, the active edges will be closed and the growth will eventually stop.

Since it is difficult to control all parameters in the growth of VG, different surface morphologies, including petal-shaped, maze-shaped, and plate-shaped, appear in the VG, while its height ranges from several hundred nanometers to several microns [14,23,24,29]. Vertical graphene is likely to develop branches with increasing height, which change the morphology of VG and affect its characteristics. For example, when VG nanosheets develop branches, mass transport channels are blocked, which reduces performance of VG-based devices, e.g., supercapacitors and gas sensors. The higher VG is preferred for VG-based supercapacitors or gas sensors, due to larger specific surface area. However, most studies have not pointed out why there are more and more branches while the height of VG increases. Hence, it is particularly important to investigate the factors that influence the height and spatial structure of VG.

Here, we study how the substrate types affect the surface morphology and vertical structure of VG. In our research, scanning electron microscopy (SEM) and Raman spectroscopy are used to characterize VG systematically from directions of surface and cross-section to show the specific influence of substrate types on VG growth, thus summarizing a VG growth rule related to substrate types and growth time. Understanding the influence of substrate types is of great help to solve the branching problem when VG increases in height.

## 2. Materials and Methods

### 2.1. Synthesis of VG

In the PECVD system (Galanz, Foshan, China) modified from a commercial microwave oven, the VGs were synthesized on Cu foils, SiO_2_/Si (90 nm), quartz, and mica using methane (CH_4_) and hydrogen (H_2_) as precursors. Prior to growth, the substrates were sonicated in an ethanol solution, and rinsed with deionized water to remove impurities from the surface of substrates. After drying with nitrogen, the substrates were placed in the center of a quartz tube in the PECVD system. Before the deposition process, the PECVD chamber was firstly cleaned by H_2_ plasma at a H_2_ flow rate of 20 sccm and a pressure of 400 Pa for 3 min to remove the oxygen and contaminants on the surface of substrates. During the growth process, the system is filled to 400 Pa with a mixture of 10 sccm CH_4_ and 10 sccm H_2_, while the base pressure is 10 Pa. After 4, 8, and 16 min depositions, the samples were naturally cooled down to room temperature under the same gas flow.

### 2.2. Characterizations of VG

The surface morphology and cross-section images of VG were observed by scanning electron microscope (SEM, Zeiss Sigma-300, Jena, Germany) with a 10-kV electron-gun voltage and 8-mm working distance. In addition, Raman spectra were recorded with a WITec alpha300 R system (Ulm, Germany) using a laser with wavelength of 488 nm at room temperature and under ambient conditions. It is noteworthy that all Raman spectra shown in this study were collected by using a 50× L objective lens for 5 s with a laser power of 0.16 mW.

## 3. Results and Discussion

**Figure 1:**
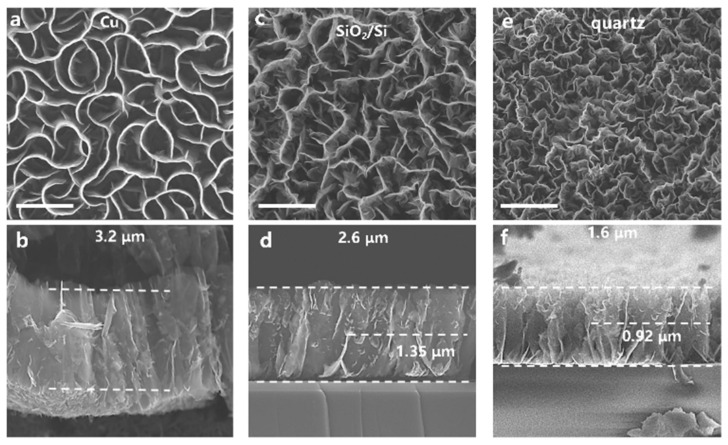
SEM images of morphology of vertical graphene (VG) on substrates with different conductive types. Top-view (**a**) and cross-sectional (**b**) images of VG on Cu; top-view (**c**) and cross-sectional (**d**) images of VG on SiO_2_/Si; and top-view (**e**) and cross-sectional (**f**) images of VG on quartz. The scale bars are 1 μm.

Figure 1 shows a SEM top view and cross-section view of VG grown on substrates of different conductivity types (Cu, SiO_2_/Si, and quartz) with the same deposition time (6 min). The VG grown on Cu is petal-shaped and relatively smooth with almost no branches, as shown in Figure 1a. The VG grown on SiO_2_/Si and quartz has a maze shape, as shown in Figure 1c,e, respectively. The overall height of VG nanosheets grown on SiO_2_/Si and quartz is smaller than that grown on Cu substrate. It can be seen from Figure 1c,d that the interfaces between VG and SiO_2_/Si or quartz are relatively abrupt, whereas the height of the smooth section of VG (on SiO_2_/Si) is higher than that of VG on quartz. After the smooth section, VG nanosheets develop many branches. Generally, the plasma sheath affects the electric field on the surface of substrates, which influences the direction and density of carbon active species during deposition. If the substrate is a conductor, the electric field changes the charge distribution on the conductor, resulting in an induced electric field in the direction perpendicular to the surface of the substrate. On the other hand, if the substrate is an insulator, the induced electric field is weaker, since there is no excessive amount of free charges in an insulator. For a semiconductor, the strength of induced electric field depends on carrier density, which normally is less than that of a conductor but larger than that of an insulator. Hence, the morphology and structural order of VG is strongly correlated with the electrical conductivity of substrates [30]. In the spatial electric field, since Cu is a good conductor, its surface electric field is the strongest, followed by that of SiO_2_/Si, and the dielectric quartz is the weakest. The surface electric field intensity determines the height at which VG nanosheets can grow smoothly in the vertical direction. Far away from the substrate surface, the electric field intensity is too weak to affect carbon ions to deposit directionally, so VG nanosheets will bend or branch.

**Figure 2:**
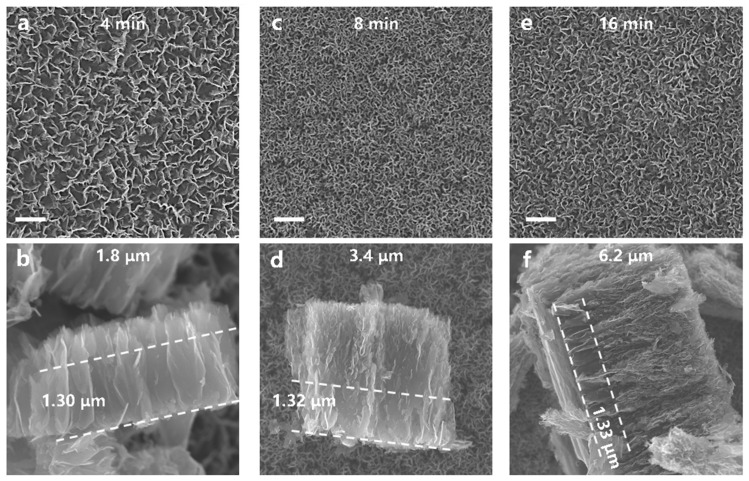
SEM images of morphologies of VG deposited on mica with different growth times. Top-view (**a**) and cross-sectional (**b**) images of VG with 4 min growth time; the total height is 1.8 μm and the height of the smooth section is 1.3 μm. Top-view (**c**) and cross-sectional (**d**) images of VG with 8 min growth time; the total height is 3.4 μm and the height of the smooth section is 1.32 μm. Top-view (**e**) and cross-sectional (**f**) images of VG with 16 min growth time; the total height is 6.2 μm and the height of the smooth section is 1.33 μm. The scale bars are 1 μm.

Figure 2 depicts SEM images of surface morphology and vertical structure of VG grown on the same substrate (mica) but with different deposition times. The surface morphology and height of VG changed with the growth time. Vertical graphene nanosheets become higher and more crowded, indicating more branches, with increasing deposition time. This means that with the same substrate, the longer the growth time, the higher the VG nanosheets with more branches. Note, the height of the smooth section in contact with the substrate remains basically unchanged. Since mica is an insulator, the surface electric field induced by the space electric field is rather weak, which leads to easier development of branches on the VG nanosheets.

**Figure 3:**
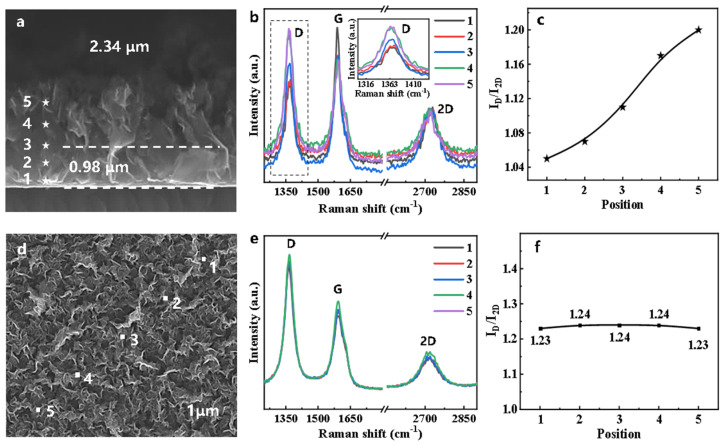
Raman characterizations of VG: (**a**) The cross−section SEM image of VG. The stars in (**a**) represent positions from which Raman spectra were collected. (**b**) Raman spectra of VG and (**c**) I_G_/I_2D_ ratios corresponding to the positions marked in (**a**). (**d**) The surface SEM image of VG. The dots in (**d**) represents positions from which Raman spectra were collected. (**e**) Raman spectra of VG and (**f**) I_G_/I_2D_ ratios corresponding to the positions in (**d**).

In order to investigate the crystalline quality of VG nanosheets, Raman spectroscopy, a powerful nondestructive analysis tool, was used to characterize the VGs. Figure 3a,d shows the typical cross-section structure and surface morphology of VG grown on SiO_2_/Si. The Raman spectra in Figure 3b were collected from the corresponding position in Figure 3a. Clear and sharp 2D peaks prove the existence of graphene, and the intensity ratio between 2D peak and G peak, I_2D_/I_G_, was approximately 0.5, suggesting that the VG consisted of few layers of graphene. The existence of the D feature indicates that there were defects in the VG nanosheets. The inset in Figure 3b is an enlarged display of the D peak, which increased when the probing area was further away from the substrate. The crystal quality of VG nanosheets was correlated with the intensity ratio of D and 2D peaks (I_D_/I_2D_) in Raman spectra. As shown in Figure 3c, the I_D_/I_2D_ first increased relatively fast, then slowed down with the increase of height. This not only shows that the crystal quality of VG nanosheets decreased with height, but also shows that the degree of disorder in VG nanosheets tended to saturate. Similarly, we also selected five points on the surface of VG to collect Raman spectra. Figure 3e shows almost overlapping Raman spectra with consistent position, shape, and intensity for all features. The I_D_/I_2D_ shown in Figure 3f is about unchanged, which suggests a uniform structure for VG on the surface. The characterization results in both vertical and surface directions show the different deposition behavior of carbon ions at different heights.

**Figure 4:**
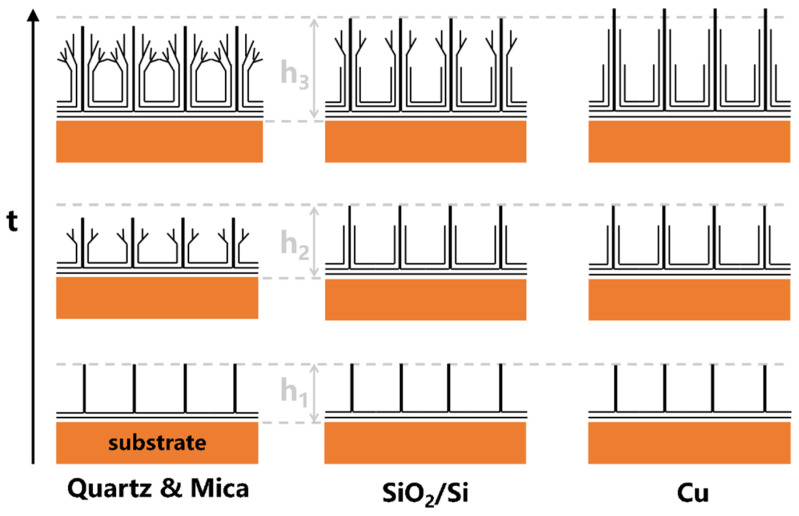
Growth pattern of VG on quartz & mica, SiO_2_/Si & Cu substrates. The ordinate represents growth time & h means total VG height (h_1_ < h_2_ < h_3_).

Based on the abovementioned characterization, the correlation between VG structure and type of substrate and deposition time was proposed and depicted in Figure 4. Since electric fields near substrate surfaces increase with increasing substrate conductivity, carbon ions tend to position in a more orderly manner, which results in fewer defects, especially for the region near the substrate surface. This leads to fewer branches in VG nanosheets and larger smooth sections near the substrate. With the increase of growth time, both the height and degree of disorder of VG nanosheets gradually increase. Note, for substrate with good electrical conductivity, carbon ions are aligned by electric field near the substrate surface to grow in a perpendicular direction to the substrate. With the decrease of electric field strength, the orderly growth of VG induced by the electric field will be overwhelmed by the random growth induced by the thermal kinetic motion of carbon ions, which results in deposition in an uncontrolled manner. This reduces the height of VG nanosheets, generates branches, and increases the degree of disorder. However, it is possible to further increase the quality and height by properly controlling the substrate conductivity and growth time or increasing the plasma power. 

Meanwhile, the plasma will induce an eddy current in the substrate, which is larger for a good conducting material, and results in a higher substrate temperature. Some literature has reported that higher substrate temperature usually leads to a better crystallinity [20,27]. This also help to produce a better quality VG. The further application of such VG is under investigation.

## 4. Conclusions

We demonstrated the electrical conductivity of substrates greatly influences the morphology and structure of VG prepared by PECVD. Fewer branches develop for VG grown on more conductive substrate. Even though the height of VG increases with increasing growth time, the number of branches also rises. The results of Raman spectra obtained at different positions suggest that the structure of VG is homogenous in the horizontal direction, but inhomogeneous in the vertical direction (e.g., more defects/branches are located at positions further away from the substrate). This suggests that tuning the strength and/or orientation of the electric field might further improve the quality of VG, and offers a novel approach for optimizing the structure of other thin films.
